# Downregulation of SREBP inhibits tumor growth and initiation by altering cellular metabolism in colon cancer

**DOI:** 10.1038/s41419-018-0330-6

**Published:** 2018-02-15

**Authors:** Yang-An Wen, Xiaopeng Xiong, Yekaterina Y. Zaytseva, Dana L. Napier, Emma Vallee, Austin T. Li, Chi Wang, Heidi L. Weiss, B. Mark Evers, Tianyan Gao

**Affiliations:** 1 Markey Cancer Center, University of Kentucky, Lexington, KY 40536-0509 USA; 2 Department of Toxicology and Cancer Biology, University of Kentucky, Lexington, KY 40536-0509 USA; 3 Paul Laurence Dunbar High School, Lexington, KY 40513 USA; 4 Department of Surgery, University of Kentucky, Lexington, KY 40536-0509 USA; 5 Department of Molecular and Cellular Biochemistry, University of Kentucky, Lexington, KY 40536-0509 USA

## Abstract

Sterol regulatory element-binding proteins (SREBPs) belong to a family of transcription factors that regulate the expression of genes required for the synthesis of fatty acids and cholesterol. Three SREBP isoforms, SREBP1a, SREBP1c, and SREBP2, have been identified in mammalian cells. SREBP1a and SREBP1c are derived from a single gene through the use of alternative transcription start sites. Here we investigated the role of SREBP-mediated lipogenesis in regulating tumor growth and initiation in colon cancer. Knockdown of either SREBP1 or SREBP2 decreased levels of fatty acids as a result of decreased expression of SREBP target genes required for lipid biosynthesis in colon cancer cells. Bioenergetic analysis revealed that silencing SREBP1 or SREBP2 expression reduced the mitochondrial respiration, glycolysis, as well as fatty acid oxidation indicating an alteration in cellular metabolism. Consequently, the rate of cell proliferation and the ability of cancer cells to form tumor spheroids in suspension culture were significantly decreased. Similar results were obtained in colon cancer cells in which the proteolytic activation of SREBP was blocked. Importantly, knockdown of either SREBP1 or SREBP2 inhibited xenograft tumor growth *in vivo* and decreased the expression of genes associated with cancer stem cells. Taken together, our findings establish the molecular basis of SREBP-dependent metabolic regulation and provide a rationale for targeting lipid biosynthesis as a promising approach in colon cancer treatment.

## Introduction

Although diverse in type and underlying genetic alterations, cancers are fundamentally a disorder of cell growth and proliferation, which requires increased cellular building blocks, such as nucleic acids, proteins, and lipids^1^. To cope with these elevated requirements cancer cells undergo major metabolic modifications^2,3^. There has been increasing interest in cancer cell metabolism as a means to understand the functional distinction between transformed and normal cells and to provide critical mechanistic insights regarding cancer development and progression^4^. Among metabolic alterations, increased de novo lipid biosynthesis has been recognized as one of the important but not well-characterized hallmarks of cancer cells^5^. Relatively few studies have rigorously examined the role of lipogenesis in promoting colorectal cancer (CRC) and how lipogenic pathways are regulated.

Sterol regulatory element-binding proteins (SREBPs) is a small family of membrane-bound, basic helix-loop-helix leucine zipper (bHLH-LZ) transcription factors that regulate the expression of genes required for the synthesis of fatty acids, triglycerides and cholesterol^6–8^. Three SREBP isoforms, SREBP1a, SREBP1c, and SREBP2, have been identified in mammalian cells that control distinct but overlapping lipogenic transcriptional programs^7–9^. A rich body of research has demonstrated that SREBP1a activates fatty acid and cholesterol synthesis, SREBP1c fatty acid synthesis, and SREBP2 cholesterol synthesis in insulin-responsive tissues such as liver and adipose tissue. The activation process of SREBPs is known to be tightly controlled by the availability of sterols^8,10^. Specifically, the newly synthesized SREBPs are expressed as inactive precursors and reside as integral trans-membrane proteins within the endoplasmic reticulum (ER) membrane where they bind to the sterol-sensing SREBP cleavage-activating protein (SCAP). When intracellular sterol concentrations are low, the SREBP/SCAP complex trafficks to the Golgi where SREBP is cleaved by site-1 and site-2 proteases and the N-terminal bHLH-LZ domain of the protein is released and translocated to the nucleus where it binds to sterol regulatory element (SRE)-sequences in the promoters of its target genes, ultimately increases sterol levels^8–11^. As a feedback mechanism to regulate sterol synthesis, cholesterol and its hydroxylated derivatives, such as 25-hydroxycholesterol (25-HC), inhibit the proteolytic cleavage and prevent the activation of SREBPs^12^. Specifically, 25-HC binds to ER anchor protein Insig to promote the formation of SCAP–Insig complex and prevent trafficking of SREBP–SCAP complex to the Golgi^13^. Moreover, fatostatin, a non-sterol-like small molecule inhibitor of SREBP, has been developed to attenuate SREBP-dependent lipogenesis by binding to SCAP to block the ER–Golgi translocation of SREBPs^14^.

In addition to their role in maintaining the homeostasis of lipid metabolism^8^, emerging evidence suggests that increased activation of SREBPs is required to sustain cancer cell proliferation. For example, activation of SREBP1 and enhanced expression of its target genes have been observed in human glioblastoma multiforme carrying activating EGFR mutations and inhibition of lipid synthesis blocks the growth of xenograft tumors derived from glioblastoma cells expressing mutant EGFR^15^. In addition, it has been shown that the expression of SREBP1 is elevated in prostate cancer patients^16^. High SREBP1 expression is positively associated with tumor metastasis and predicts poor prognosis in breast cancer patients^17^. Moreover, activation of SREBP as a result of mTORC1 activation downstream of oncogenic PI3K and KRAS signaling has been shown to promote breast cancer cell growth and proliferation^18^. Consistently, the expression of SREBP target genes, such as fatty acid synthase (FASN) and steroyl-CoA desaturase (SCD), are also increased in a variety of human cancers^1,19–21^. However, the function of SREBPs in human CRC remains to be fully elucidated.

In this study, we investigated the functional importance of SREBP-mediated lipogenesis in regulating cellular metabolism and tumor growth in colon cancer. Inhibiting lipid biosynthesis by silencing SREBP1, SREBP2 or their regulator SCAP reduced the rate of cell proliferation as well as tumor-initiating potential *in vitro*. Moreover, downregulation of SREBP expression or inhibition of SREBP activation markedly altered cellular metabolic pathways. Importantly, knockdown of SREBPs inhibited xenograft tumor growth and initiation *in vivo*. Taken together, our findings demonstrate that SREBPs have an important role in promoting tumorigenesis in colon cancer.

## Results

### Knockdown of SREBP1 or SREBP2 decreases cell proliferation and spheroid formation in colon cancer cells

**Fig. 1:**
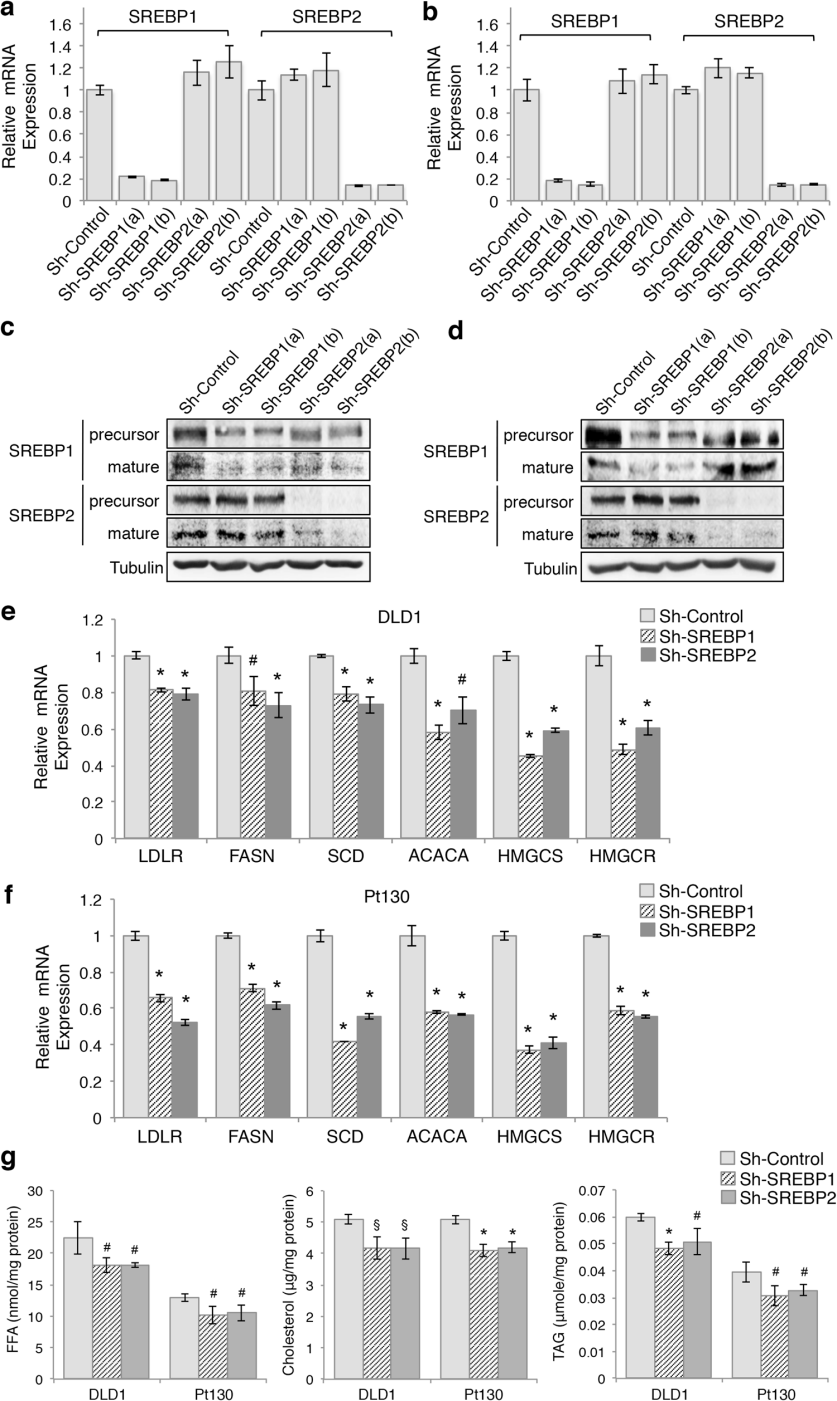
Knockdown of SREBP1 or SREBP2 inhibits lipogenesis in colon cancer cells. **a**, **b** The real-time PCR analysis of SREBP1 and SREBP2 expression in control and knockdown DLD1 (**a**) and Pt130 (**b**) cells. Two different shRNA targeting sequences were used for knocking down SREBP1 or SREBP2. **c**, **d** The expression of SREBP1 and SREBP2 proteins were downregulated in stable DLD1 (**c**) and Pt130 (**d**) knockdown cells. Protein lysates from control and knockdown cells were analyzed by western blot. Both the precursor and mature forms of SREBP1 and SREBP2 proteins were detected. **e**, **f** Knockdown of SREBP1 and SREBP2 decreased the expression of downstream genes related to fatty acid synthesis and metabolism in DLD1 (**e**) and Pt130 (**f**) cells. Data represent the mean ± SD (*p < 0.001 and ^#^*p* < 0.05 compared to sh-Control). **g** Knockdown of SREBP1 or SREBP2 decreased lipogenesis in colon cancer cells. Total cellular lipids were extracted from control and SREBP knockdown DLD1 and Pt130 cells and the levels of free fatty acids, cholesterol, and triglyceride were measured and normalized to the amount of total proteins. Data represent the mean ± SD (*p < 0.001, ^§^*p* < 0.01, and ^#^*p* < 0.05 compared to sh-Control)

To begin to elucidate the role of SREBP-mediated lipid biosynthesis in colon cancer, we generated multiple SREBP1 and SREBP2 knockdown cell lines using two different lentivirus encoded shRNAs (Fig. 1a, b). Notably, both SREBP1-specific shRNAs target common regions in the SREBP1 gene shared by SREBP1a and SREBP1c isoforms. Downregulation of both the precursor and mature forms of SREBP1 and SREBP2 protein was confirmed by western blot analysis in corresponding knockdown cell lines (Fig. 1c, d). Consistent with their transcription factor function, silencing SREBPs led to a significant decrease in the expression of target genes in DLD1 and patient-derived Pt130 colon cancer cells (Fig. 1e, f). The target genes of SREBPs tested here are known to involved in cholesterol uptake and biosynthesis (including LDR receptor (*LDLR*), HMG-CoA reductase (*HMGCR*), and HMG-CoA synthase (*HMGCS*)) as well as fatty acid synthesis (including fatty acid synthase (*FASN*), acetyl-CoA carboxylase (*ACACA*), and stearoyl CoA desaturase (*SCD*)). Similar effects on decreasing target gene expression were observed in HCT116 cells (Supplementary Figure S1). As a consequence, intracellular levels of free fatty acids (FFA), cholesterol and triglycerides (TAG) were significantly decreased in SREBP knockdown cells (Fig. 1g).

**Fig. 2:**
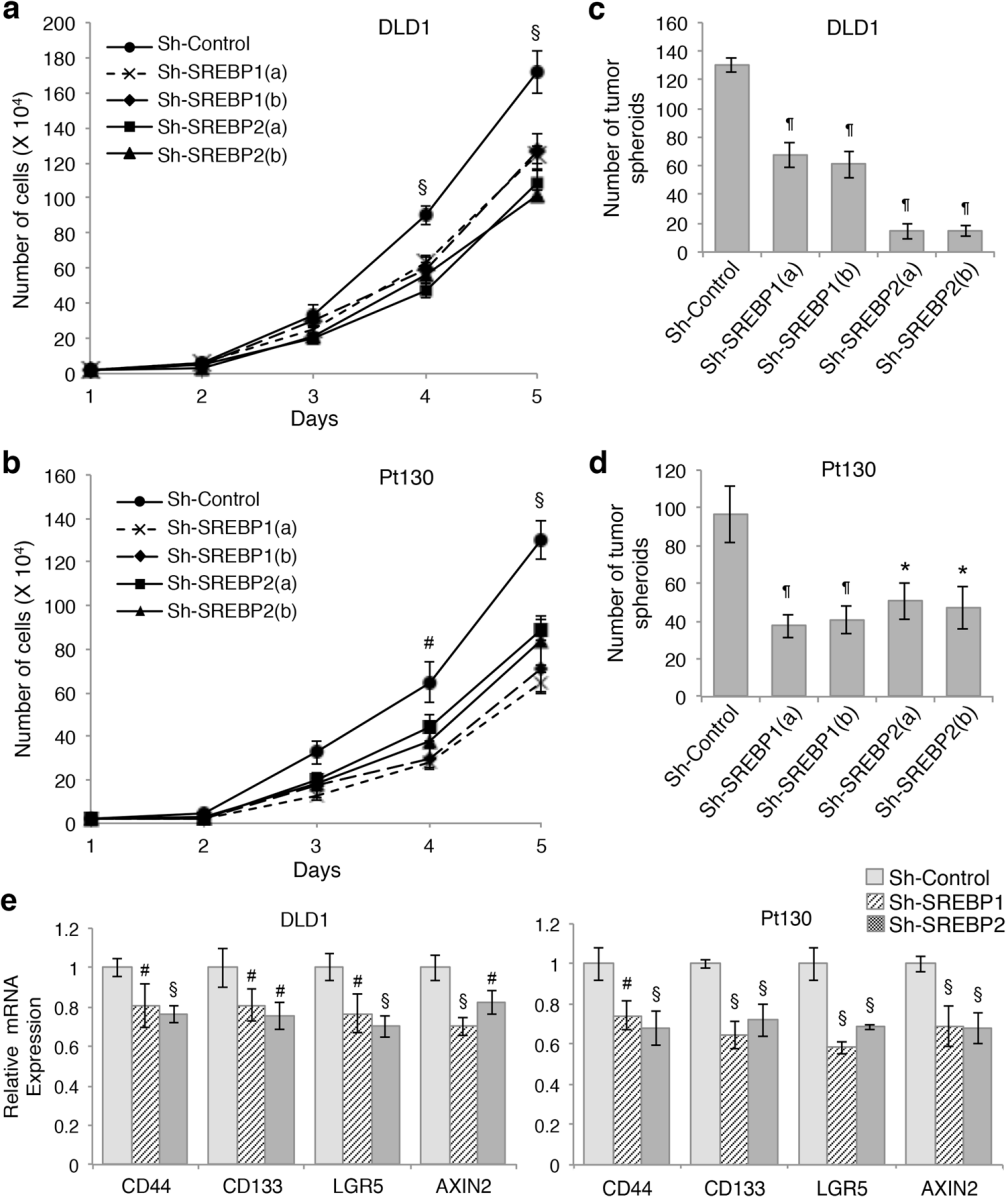
Knockdown of SREBP1 or SREBP2 inhibits cell proliferation and tumor spheroid formation in colon cancer cells. **a**, **b** Knockdown of SREBP1 or SREBP2 decreased the rate of proliferation in DLD1 (**a**) and Pt130 (**b**) cells. Equal number of control and SREBP knockdown cells were allowed to grow for 4 days and the number of cells were counted each day. Data represent the mean ± SD (^§^*p* < 0.01 and ^#^*p* < 0.05 compared to sh-Control). **c**, **d** Knockdown of SREBP1 or SRBEP-2 decreased the formation of tumor spheroids in the stem cell suspension medium. Control and SREBP knockdown DLD1 (**c**) and Pt130 (**d**) cells were seeded as single cells in the stem cell suspension medium and the number of colonies formed was determined after 6 days. Data represent the mean ± SD (^¶^*p* < 0.0001 and *p < 0.001 compared to sh-Control). **e** Knockdown of SREBP1 or SRBEP2 reduced the expression of genes associated with colon cancer stem cells. The relative expression of *CD44*, *CD133*, *LGR5*, and *AXIN2* mRNA was determined using real-time PCR in control and SREBP knockdown DLD1 and Pt130 cells. Data represent the mean ± SD (^§^*p* < 0.01 and ^#^*p* < 0.05 compared to sh-Control)

To investigate the functional effect of silencing SREBPs, we monitored the rate of cell proliferation in control and SREBP1 or SREBP2 knockdown DLD1 and Pt130 cells. Silencing either SREBP1 or SREBP2 was sufficient to reduce cell growth in both cell lines when compared to control cells (Fig. 2a, b). Similar results were obtained in SREBP1 and SREBP2 knockdown HCT116 cells (Supplementary Figure S2a). Furthermore, we determined if SREBP-dependent lipogenesis is required for maintaining the tumor initiation potential of colon cancer cells. To this end, single cells of control and SREBP1 or SREBP2 knockdown DLD1 and Pt130 cells were cultured in suspension stem cell medium, a condition known to enrich tumor-initiating cells^22^, and numbers of spheroids (colonies) formed were counted after 6 days. While approximately 10% of control DLD1 and Pt130 cells formed tumor spheroids in suspension, the numbers of spheroids were significantly decreased in SREBP1 and SREBP2 knockdown cells suggesting inhibition of colony formation (Fig. 2c, d). In addition, knockdown of SREBP1 and SREBP2 resulted in a significant decrease in the number of tumor spheroids formed in HCT116 cells (Supplementary Figure S2b). Consistently, qPCR analysis revealed that the expression of genes associated with colon cancer stem cells, including *CD44, CD133, LGR5*, and *AXIN2*, was reduced in SREBP1 and SREBP2 knockdown DLD1 and Pt130 cells (Fig. 2e). Taken together, these data suggest that SREBP1-mediated and SREBP2-mediated lipid biosynthesis has an important role in supporting cell proliferation and tumor initiation potential of colon cancer cells.

### Inhibition of proteolytic processing of SREBPs decreases cell proliferation and spheroid formation in colon cancer cells

**Fig. 3:**
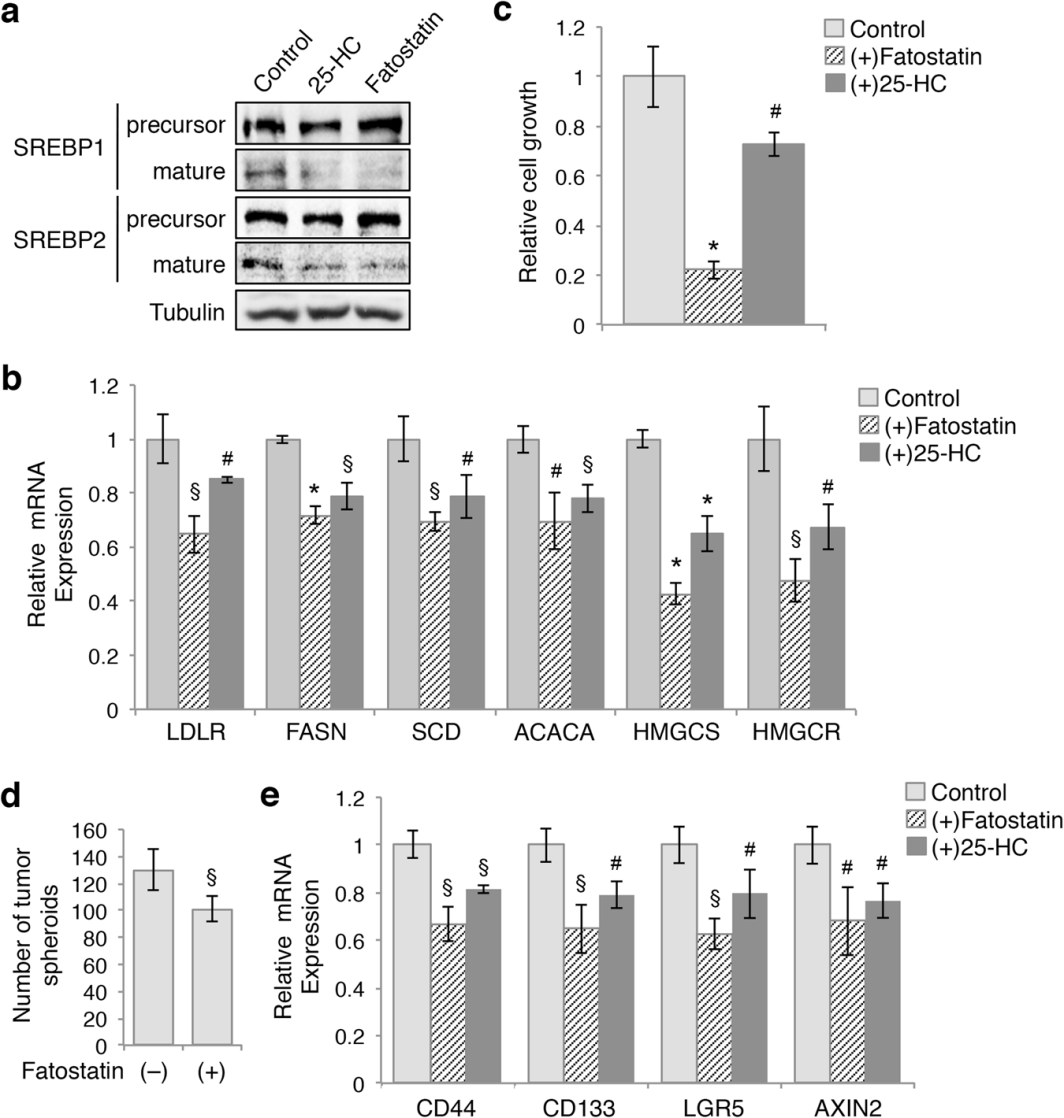
Inhibition of SREBP activation decreases cell survival and tumor spheroid formation in colon cancer cells. **a** Treating cells with 25-HC (10 μg/ml) or fatostatin (30 μM) blocks the proteolytic processing of SREBPs in DLD1 cells. Protein lysates from control and treated cells were analyzed for the expression of SREPBs by western blot. **b** Inhibition of SREBPs decreased the cell survival. DLD1 cells were grown in the presence or absence of fatostatin and 25-HC for 72 h and numbers of cells was counted. The relative cell growth was calculated by normalizing the number of cells to the control condition. Data represent the mean ± SD (*p < 0.0001 and ^#^*p* < 0.05 compared to control untreated cells). **c** Inhibition of SREBPs decreased the expression of downstream genes related to fatty acid synthesis and metabolism in DLD1 cells. Data represent the mean ± SD (*p < 0.001, ^§^*p* < 0.01, and ^#^*p* < 0.05 compared to control untreated cells). **d** Fatostatin treatment inhibited the formation of tumor spheroids in the stem cell suspension medium. DLD1 cells were seeded as single cells in the stem cell suspension medium in the presence of absence of fatostatin and the number of colonies formed was determined after 6 days. Data represent the mean ± SD (^§^*p* < 0.01 compared to untreated cells). **e** Inhibition of SREBPs reduced the expression of genes associated with colon cancer stem cells. The relative expression of *CD44*, *CD133*, *LGR5*, and *AXIN2* mRNA was determined using real-time PCR in control and fatostatin or 25-HC treated DLD1 cells. Data represent the mean ± SD (^§^*p* < 0.01 and ^#^*p* < 0.05 compared to control untreated cells)

We next determined whether inhibition of SREBP activation by blocking proteolytic cleavage has similar effects as silencing SREBP directly. Consistent with previous reports, treating cells with 25-HC or fatostatin markedly decreased the levels of mature (transcriptionally active) form of SREBP1 and SREBP2 in DLD1 cells as shown by western blot analysis (Fig. 3a). Similar as those observed in SREBP1 and SREBP2 knockdown cells, 25-HC-induced and fatostatin-induced inhibition of SREBPs activation significantly reduced the expression of downstream target genes important for lipid biogenesis (Fig. 3b). To determine the functional effect of SREBP inhibition, DLD1 cells were cultured in the presence of 25-HC or fatostatin. We found that both fatostatin and 25-HC significantly inhibited cell growth, however, fatostatin showed a strong inhibitory effect (Fig. 3c). This is likely due to the fact that fatostatin binds and inhibits SCAP-mediated activation of SREBP in a sterol-independent manner, whereas 25-HC competes directly with sterols in cell culture media^14^. Furthermore, results from spheroid formation experiments showed that fatostatin treatment reduced the number of tumor spheroids formed in suspension (Fig. 3d). Interestingly, the 25-HC treatment was unable to inhibit tumor spheroid formation (data not shown). As the suspension medium used in this assay contains high levels of bovine serum albumin (BSA), BSA-bound lipids may attenuate the 25-HC-mediated inhibitory effect. In addition, 25-HC-induced and fatostatin-induced inhibition of SREBP activation decreased expression of genes associated with cancer stem cells (Fig. 3e).

To further confirm the functional importance of de novo lipid biosynthesis in colon cancer, we silenced the expression of SCAP, a sterol-sensing SCAP, in DLD1 and Pt130 cells using lentivirus-mediated RNAi. Consistent with its role in facilitating ER–Golgi translocation and proteolytic cleavage of SREBPs, downregulation of SCAP resulted in a similar decrease in the expression of genes required for lipid biosynthesis (Supplemental Figure S3a). In addition, knockdown of SCAP inhibited cell proliferation in both colon cancer cell lines (Supplemental Figure S3b). Furthermore, silencing SCAP significantly inhibited tumor spheroid formation and decreased expression of genes associated with cancer stem cells (Supplemental Figure S3c, d). Collectively, these results indicate that inhibition of lipid biosynthesis, via downregulation of SREBPs or inhibition of proteolytic cleavage of SREBPs, significantly inhibited cell growth and reduced cancer stem cell properties.

### Downregulation of SREBP alters cellular metabolism

**Fig. 4:**
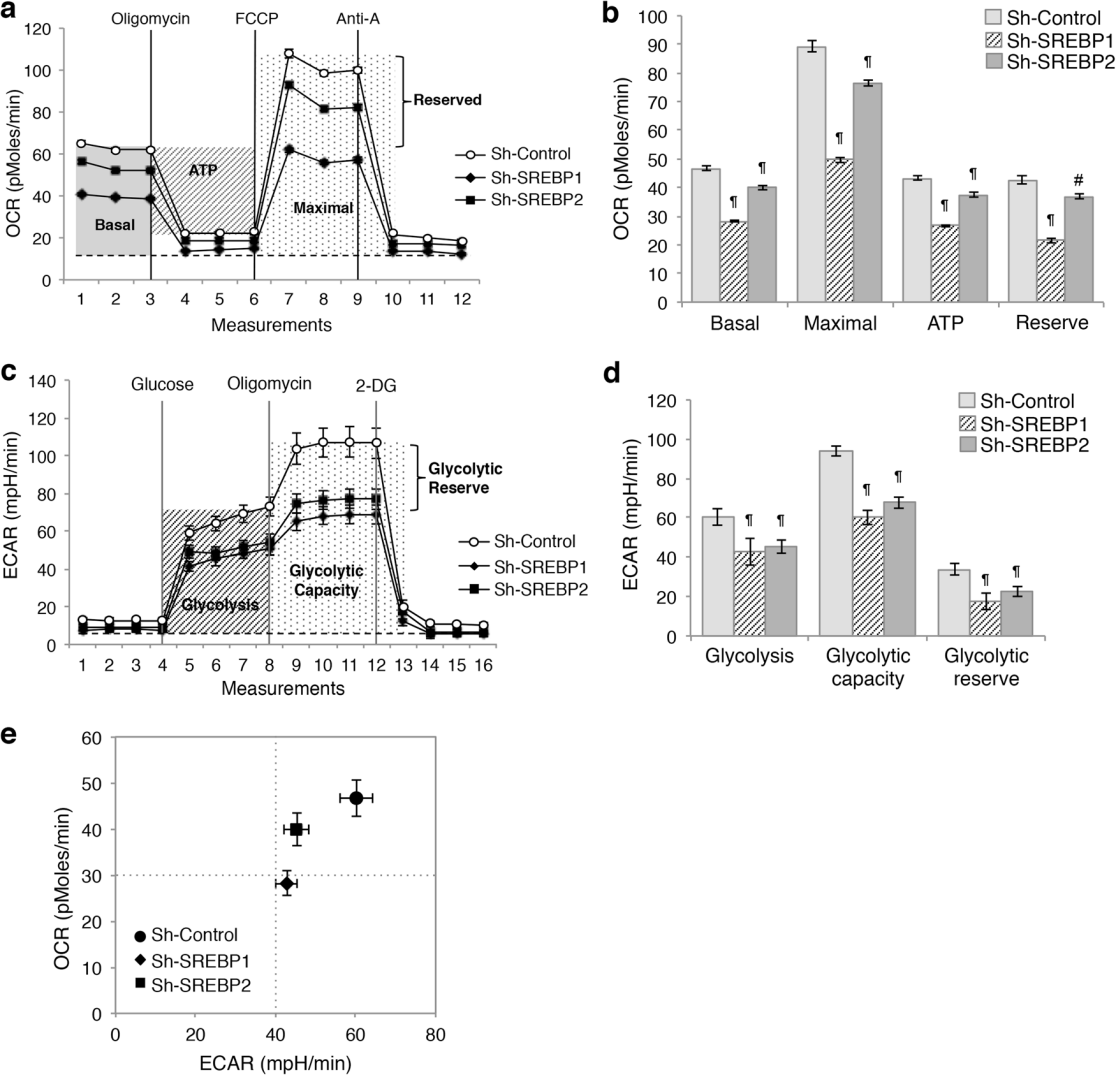
Knockdown of SREBP1 or SREBP2 deregulates glycolysis and mitochondrial respiration in colon cancer cells. **a** Representative OCR measurements obtained from the Mito tress test performed in control (sh-Control) and SREBP knockdown (sh-SREBP1 and sh-SREBP2) Pt130 cells using the Seahorse XF96 Extracellular Flux analyzer. Oligomycin, FCCP, and antimycin A (Anti-A) were added at the indicated points. The shaded areas indicate the OCR levels associated with basal and maximal respiration, ATP turnover and reserved capacity, respectively. **b** Experiments as shown in **a** were quantified and the relative levels of OCR associated with basal and maximal respiration, ATP turnover and reserved capacity were calculated. Data represent the mean ± SEM (^¶^*p* < 0.0001 and ^#^*p* < 0.05 compared to Sh-Control). **c** Representative ECAR measurements obtained from the glycolysis stress test performed in control and SREBP knockdown Pt130 cells using the Seahorse XF96 Extracellular Flux analyzer. Glucose, oligomycin, and 2-deoxyglucose (2-DG) were added at the indicated points. The shaded areas indicate the ECAR levels associated with glycolysis, glycolytic capacity, and glycolytic reserve, respectively. **d** Experiments as shown in **c** were quantified and ECAR associated with glycolysis, glycolytic capacity, and glycolytic reserve was calculated. Data represent the mean ± SEM (^¶^*p* < 0.0001 compared to Sh-Control). **e** Knockdown of SREBP resulted in metabolic shifts in Pt130 cells. The energetic map of control and SREBP knockdown Pt130 cells was based on the measurements obtained in **a** and **c**. The OCR and ECAR data shown in the map represent the OCR of basal respiration from the Mito stress test and the glycolysis-related ECAR from the glycolysis stress test, respectively (data represent the mean ± SEM)

Accumulating evidence suggests that alterations in lipid metabolism contribute to overall metabolic reprogramming in cancer cells^1,5,23^. To better understand the mechanism underlying SREBP-mediated regulation of cell proliferation and stem cell properties, we determined the functional effect of SREBP-loss on modulating other metabolic pathways in colon cancer cells. To this end, we performed Mito Stress Tests using Seahorse XF96 Extracellular Flux Analyzer. Results showed that oxygen consumption rates (OCR) associated with both basal and maximal mitochondrial respiration was significantly decreased in SREBP1 and SREBP2 knockdown cells (Fig. 4a, b). In addition, mitochondrial respiration used for ATP production and reserved mitochondrial capacities were reduced in SREBP knockdown cells (Fig. 4a, b). Moreover, control, SREBP1 and SREBP2 knockdown cells were subjected to seahorse glycolysis stress tests to evaluate the glycolysis phenotype. Interestingly, silencing SREBP1 or SREBP2 significantly decreased the extracellular acidification rate (ECAR) associated with glycolysis, glycolytic capacity and glycolytic reserves in Pt130 cells (Fig. 4c, d). Similar results were obtained in SREBP1 and SREBP2 knockdown DLD1 cells (data not shown). Furthermore, bioenergetic measurements of relative utilization of mitochondrial respiration and glycolysis under basal conditions confirmed that knockdown of either SREBP reduced both the glycolytic potential and the mitochondria-mediated oxidative phosphorylation in colon cancer cells (Fig. 4e). These results suggest that SREBP-dependent lipid biogenesis has an important role in maintaining overall metabolic activity in cancer cells.

**Fig. 5:**
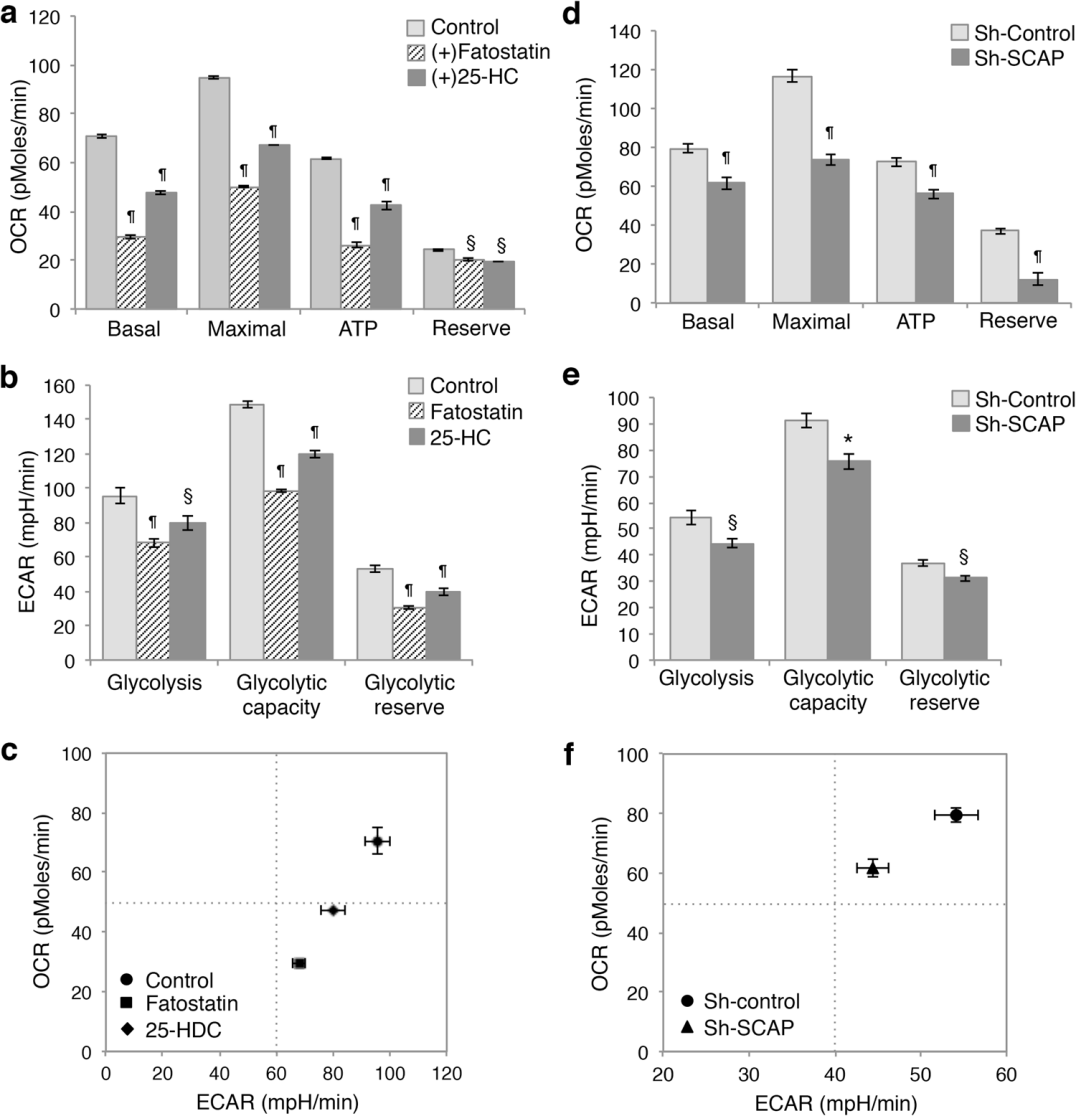
Inhibition of SREBP proteolytic processing alters cellular metabolism in colon cancer cells. Control and fatostatin or 25-HC treated DLD1 cells were subjected to metabolic analysis using the Seahorse XF96 Extracellular Flux analyzer as described in Fig. 4. **a** The relative levels of OCR associated with basal and maximal respiration, ATP turnover and reserved capacity were calculated based on the measurements obtained in the Mito stress test. Data represent the mean ± SEM (^¶^*p* < 0.0001 and ^§^*p* < 0.01 compared to control untreated cells). **b** The levels of ECAR associated with glycolysis, glycolytic capacity, and glycolytic reserve were calculated based on the measurements obtained in the glycolysis stress test. Data represent the mean ± SEM (^¶^*p* < 0.0001 and ^§^*p* < 0.01 compared to control untreated cells). **c** The energetic map of control and treated DLD1 cells was based on the measurements obtained in **a** and **c**. The OCR and ECAR data shown in the map represent the OCR of basal respiration from the Mito stress test and the glycolysis-related ECAR from the glycolysis stress test, respectively (data represent the mean ± SEM). **d** Control and SCAP knockdown Pt130 cells were subjected to metabolic analysis using the Seahorse XF96 Extracellular Flux analyzer. The relative levels of OCR associated with basal and maximal respiration, ATP turnover and reserved capacity were calculated based on the measurements obtained in the Mito stress test. Data represent the mean ± SEM (^¶^*p* < 0.0001 compared to Sh-Control). **e** The levels of ECAR associated with glycolysis, glycolytic capacity and glycolytic reserve was calculated based on the measurements obtained in the glycolysis stress test. Data represent the mean ± SEM (*p < 0.001 and ^§^*p* < 0.01 compared to Sh-Control). **f** The energetic map of control and SCAP knockdown Pt130 cells was derived from the measurements obtained in **d** and **e**. The OCR and ECAR data shown in the map represent the OCR of basal respiration from the Mito stress test and the glycolysis-related ECAR from the glycolysis stress test, respectively (data represent the mean ± SEM)

To confirm the effects of SRBEP downregulation on modulating cellular metabolism, we assessed the levels of mitochondrial respiration and glycolysis in cells treated with fatostatin or 25-HC. Bioenergetic measurements obtained using the Seahorse XF Analyzer showed that inhibition of SREBP activation by treating cells with fatostatin or 25-HC decreased both basal and maximal mitochondrial respiration as well as mitochondrial respiration associated with ATP production and reserved capacities in DLD1 cells (Fig. 5a). In addition, fatostatin or 25-HC treatment significantly reduced levels of glycolysis, glycolytic capacity, and glycolytic reserve (Fig. 5b). The cell bioenergetic phenotype analysis revealed that inhibition of lipid biogenesis resulted in a decrease in both mitochondrial oxidative phosphorylation and glycolysis (Fig. 5c). Similar results were obtained in SCAP knockdown Pt130 cells (Fig. 5e, f). Furthermore, we determined whether SREBP expression is associated with cellular metabolic pathways by analyzing the Cancer Genome Atlas (TCGA) CRC gene expression data. Indeed, results from gene set enrichment analysis (GSEA) revealed that both SREBP1 (gene name: *SREBF1*) and SREBP2 (gene name: *SREBF2*) expression was positively associated with not only lipid biosynthesis pathways but also glucose metabolism and TCA cycle in CRC patients (Supplemental Figure S4). Taken together, our results indicate that the lipid biogenesis pathway is functionally connected with glycolysis and mitochondrial-dependent metabolic pathways in CRC.

### Downregulation of lipid biogenesis decreases fatty acid oxidation

**Fig. 6:**
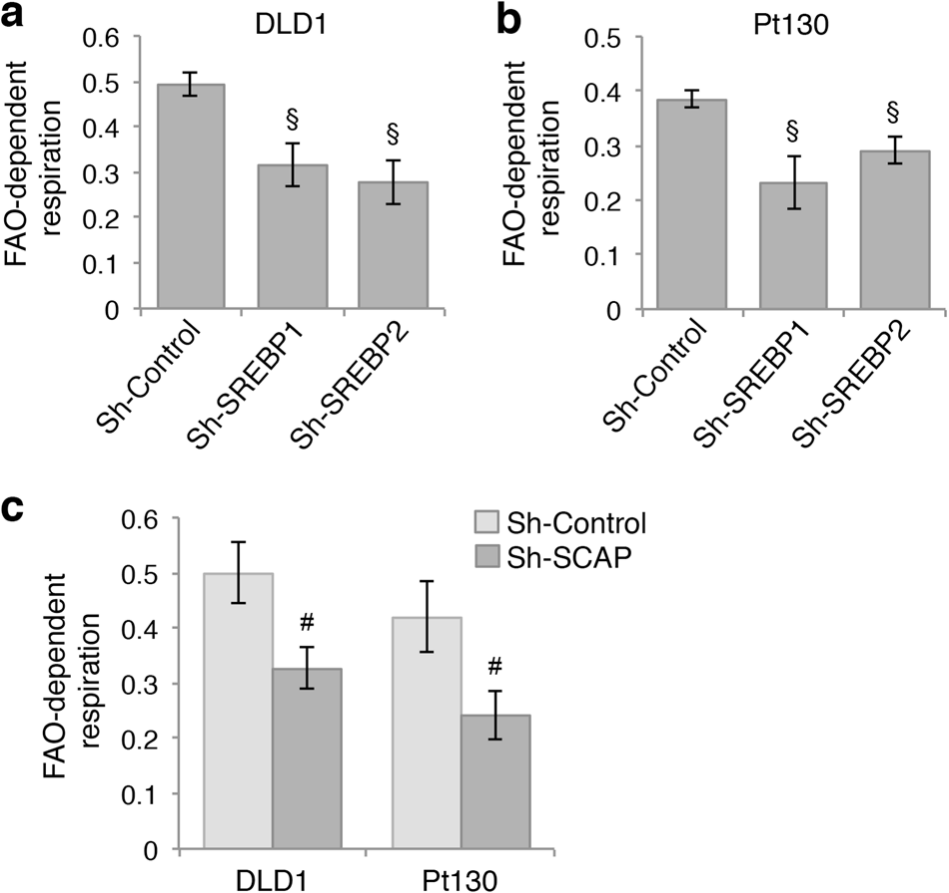
Downregulation of SREBP decreases fatty acid oxidation in colon cancer cells. **a**, **b** Knockdown of SREBP1 and SREBP2 reduced fatty acid oxidation (FAO) in DLD1 (**a**) and Pt130 (**b**) cells. The rate of cellular FAO was determined by subjecting control and SREBP knockdown cells to the FAO assays using the Seahorse XF96 Extracellular Flux analyzer. Data represent the mean ± SEM (^§^*p* < 0.01 compared to Sh-Control). **c** Knockdown of SCAP reduced FAO in DLD1 and Pt130 cells. The rate of cellular FAO was determined using Seahorse FAO assays. Data represent the mean ± SEM (^#^*p* < 0.05, compared to Sh-Control)

In normal insulin-responsive tissues, decreased fatty acid synthesis is often coupled with increased fatty acid oxidation (FAO). Here, we determined the effect of knocking down SREBPs on FAO in colon cancer cells. The FAO assays were performed to measure the OCR levels in the presence or absence of etomoxir (ETO), a carnitine palmitoyltransferase-1 (CPT-1) inhibitor, using Seahorse XF Analyzer. As no exogenous fatty acids were added in the assays, the differences in the OCR measurements were used to determine the utilization of endogenous fatty acids. Intriguingly, results obtained in control and SREBP1 and SREBP2 knockdown DLD1 and Pt130 cells showed that silencing either SREBP significantly reduced the levels of FAO in both cell lines (Fig. 6a, b). Similarly, knockdown of SCAP decreased the levels of FAO in both DLD1 and Pt130 cells (Fig. 6c). In supporting a functional connection between SREBP-mediated lipid biosynthesis and FAO in cancer cells, results from our GSEA study showed that the expression of SREBP1 and SREBP2 was positively correlated with the fatty acid metabolism pathway in CRC patient samples (Supplemental Figure S4). Taken together, these results suggest that inhibition of SREBP-mediated lipogenesis not only reduces the need for glycolysis and mitochondrial respiration but also decreases the rate of FAO in colon cancer cells.

### Silencing SREBP inhibits xenograft tumor growth in vivo

**Fig. 7:**
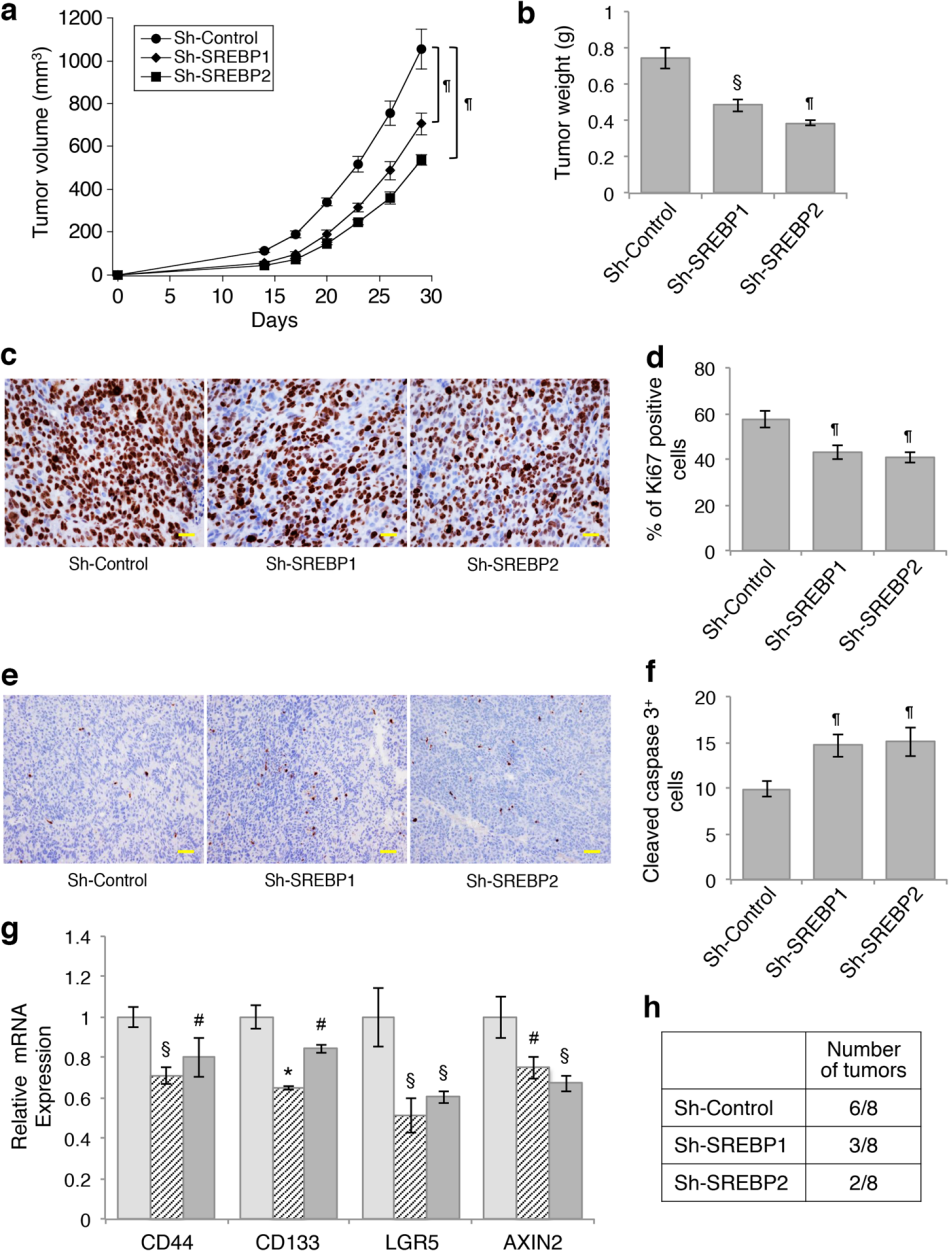
Knockdown of SREBP inhibits xenograft tumor growth and tumor initiation. **a** Control, SREBP1, and SREBP2 knockdown Pt130 cells were injected subcutaneously into NSG mice. The size of the tumors was measured every 3 days starting at day 14. Data represent the mean ± SEM (^¶^*p* < 0.0001 compared to Sh-Control group). **b** On day 29, tumors were excised and weighted. Data represent the mean ± SEM (^¶^*p* < 0.0001 and ^§^*p* < 0.01 compared to Sh-Control group). **c** Detection of proliferating cells in tumors with IHC staining using the anti-Ki67 antibody. Scale bar, 25 μm. **d** Numbers of Ki67-positive cells were quantified in 500 randomly chosen tumor cells. Data in the graph represent the mean ± SD (^¶^*p* < 0.0001 compared to Sh-Control group). **e** Detection of apoptotic cells in tumors with IHC staining using the anti-cleaved caspase-3 antibody. Scale bar, 25 μm. **f** Numbers of cleaved caspase-3-positive cells were quantified in 500 randomly chosen tumor cells. Data in the graph represent the mean ± SD (^¶^*p* < 0.0001 compared to Sh-Control group). **g** Knockdown of SREBP reduced the expression of genes associated with colon cancer stem cells. The relative expression of *CD44*, *CD133*, *LGR5*, and *AXIN2* mRNA was determined using real-time PCR in tumors derived from control, SREBP1 and SREBP2 knockdown Pt130 cells. Data represent the mean ± SD (*p < 0.001, ^§^*p* < 0.01, and ^#^*p* < 0.05 compared to Sh-Control group). **h** Tumor initiation experiments were performed using control, SREBP1, and SREBP2 knockdown Pt130 cells. The cells were mixed with Matrigel and subcutaneously inoculated into NSG mice at 1000 cells per site and total of eight injections were used for each cell line. The number of tumors formed was determined 3 months post inoculation.

Given that inhibition of lipid biosynthesis by silencing SREBPs decreases cell proliferation and stemness in colon cancer cells, we next investigated the functional effects of SREBP-mediated lipid biosynthesis in controlling tumor growth *in vivo*. To this end, control and SREBP1- or SREBP2 knockdown Pt130 cells were injected subcutaneously into NSG mice and the tumorigenesis process was monitored over 28 days. We found that silencing SREBP1 or SREBP2 significantly reduced the rate of tumor growth over the follow-up period and decreased the average weight of tumors (Fig. 7a, b). In addition, tumor sections were stained with the Ki67 and cleaved caspase-3 antibodies to determine the rate of cell proliferation and apoptosis, respectively. IHC staining results showed that the numbers of Ki67-positive cells were significantly decreased in SREBP1 and SREBP2 knockdown tumors indicating decreased cell proliferation (Fig. 7c, d). In contrast, the numbers of cleaved caspase-3-positive cells were increased indicating elevated levels of apoptosis (Fig. 7e, f), Consistent with the results obtained in cultured cells, the expression of genes associated with cancer stem cells, including *CD44*, *CD133*, *LGR5*, and *AXIN2*, was significantly decreased in SREBP1 and SREBP2 knockdown tumors (Fig. 7g). Furthermore, to confirm the effect of SREBP downregulation on reducing the stemness of colon cancer cells, we performed tumor initiation experiments by injecting control and SREBP1 and SREBP2 knockdown Pt130 cells at 1000 cells per site in NSG mice. The number of tumors formed was determined after 3 months. While control cells were able to form tumors at 6 out of 8 injection site, the tumor forming ability of SREBP1 and SREBP2 knockdown cells was decreased (Fig. 7h), suggesting that silencing SREBPs inhibits tumor initiation. Collectively, our results demonstrate that SREBP-dependent lipid biosynthesis promotes colon cancer tumorigenesis.

## Discussion

Cancer cells are rapidly dividing cells that have increased demands for energy and macromolecules. Emerging evidence suggests that the primary functions of activated oncogenes and inactivated tumor suppressors are to rewire cellular metabolic pathways in order to drive tumorigenesis^24^. Whereas most normal human cells utilize exogenous sources of fatty acids, tumors often exhibit a shift toward de novo lipid biosynthesis, despite the availability of extracellular lipids^5,25,26^. Here we show that inhibition of lipid biosynthesis by knocking down SREBP1 or SREBP2 significantly inhibits cell proliferation *in vitro* and tumorigenesis *in vivo* as a result of decreased fatty acid and cholesterol levels in colon cancer cells. In addition, silencing SREBP1 or SREBP2 expression in both established cancer cell lines and patient-derived cancer cells results in an overall shift in cellular metabolism as indicated by reduced levels of glycolysis, mitochondrial respiration as well as FAO. Importantly, knockdown of SREBPs inhibits the tumor initiation capacity of colon cancer cells and reduces the expression of cancer stem cell-associated genes. Furthermore, blocking the proteolytic processing of SREBP by treating cell with inhibitors or silencing SCAP, results in a similar inhibition of cell proliferation and alteration of cellular metabolism, thus confirming the functional importance of SREBP-dependent lipid biosynthesis. Taken together, results from our studies establish a role of SREBPs in promoting tumor growth and initiation in colon cancer.

Although it has been suggested that SREBP1 controls fatty acid and cholesterol synthesis whereas SREBP2 preferentially activates cholesterol synthesis^9^, genes related to fatty acid and cholesterol synthesis are similarly downregulated in SREBP1 and SREBP2 knockdown colon cancer cells and the functional outcomes are mostly comparable upon silencing either isoforms. One exception is that silencing SREBP1 has a stronger effect on reducing mitochondrial respiration compared to SREBP2. It is of particular interest to investigate if SREBP1 regulates expression of genes that are coupled directly with mitochondrial functions in future studies. Previous studies on defining the specificity of SREBP isoforms are generally conducted in the context of liver metabolism by overexpressing SREBP^27,28^. Our findings here suggest that SREBP1 and SREBP2 may have overlapping functions in colon cancer cells.

As a part of metabolic reprogramming, altered lipid metabolism has been increasingly recognized as an important hallmark of cancer cells^1,2^. In normal insulin-responsive tissues, such as liver and adipose tissue, decreased the cellular FAO can lead to increased production and accumulation of fatty acids^29^. Intriguingly, we find that inhibition of SREBP-mediated lipid biosynthesis reduces the mitochondrial activity associated with both glycolysis and FAO in colon cancer cells. These results suggest that levels of glucose metabolism are dictated by the need for lipid production in cancer cells, and fatty acids derived from de novo lipid biosynthesis are used to fuel tumor growth. Collectively, our findings highlight the significance of metabolic flexibility that adapted by cancer cells to fulfill the energy requirement for proliferation and survival. However, additional studies are needed to elucidate the molecular mechanism that allows the coupling of lipogenesis with FAO and other metabolic pathways in cancer cells. Recent studies using genome-wide ChIP-Seq approach have identified previously unknown target genes of SREBP1 and SREBP2 that are beyond lipid biosynthesis regulation^30,31^. For example, SREBP2 has been shown to directly activates autophagy related genes under sterol starvation condition^31^. In addition, it has been shown that inhibition of SREBP activation prevents cytokine-induced metabolic reprogramming in NK cells^32^. Thus, lipid biosynthesis-independent functions of SREBPs may also contribute to the regulation of cellular metabolism in colon cancer cells.

Given the notion that elevated lipid biosynthesis promotes tumorigenesis and tumor progression, targeting de novo lipogenesis represents an attractive option in developing new therapeutic agents in cancer treatment^33^. Indeed, it has been shown that depletion of SREBP1 and SREBP2 expression induces ER-stress, accumulation of reactive oxygen species and apoptosis in breast cancer and glioblastoma cells^34^. Downregulation of SREBP1 expression inhibits cell growth, migration and invasion and induces apoptosis in ovarian cancer cells^35^. Furthermore, fatostatin suppresses xenograft tumor growth of prostate and breast cancer cells^36,37^. In our effort to determine the levels of SREBP1 and SREBP2 expression in human CRC, we performed bioinformatics analysis using the Cancer Genome Atlas (TCGA) gene expression database. However, we did not find any significant increase in SREBP1 or SREBP2 expression in CRC patient samples compared to normal controls (data not shown). As the RAS/RAF and PI3K/AKT pathway are commonly activated in CRC, the activity of SREBPs may be increased via a post-translational mechanism downstream of Akt/mTOR rather than at the transcript level^11,15,18^.

Our study provides initial evidence that SREBP-mediated tumor promoting effects may be partially blocked by fatostatin. However, the overall anti-tumor effect of SREBP inhibition likely depends on the availability of exogenous lipids and the activation status of lipogenic signaling in cancer cells^18,33,34^. It remains an open question which types of cancer (or which cancer patients) are most likely to respond to therapies targeting de novo lipid biosynthesis pathways. Interestingly, increased lipid droplet formation has been observed in a subpopulation of colon cancer cells that express CD133, a putative marker of cancer stem cells, suggesting that cancer stem cells may maintain a higher level of lipid biosynthesis^38^. In addition, recent studies indicate that leukemia stem cells preferentially rely on FAO for the maintenance of stem cell properties^23,39^. We show here that silencing SREBP reduces FAO and the “stemness” of colon cancer cells as indicated by decreased colony formation ability *in vitro* and tumor initiation *in vivo*. Therefore, inhibition of lipid biosynthesis may provide an effective approach for targeting cancer stem cells.

In summary, results from our study demonstrate the functional importance of SREBP-mediated lipid biosynthesis in colon cancer. Future studies are needed to further explore the molecular mechanism by which lipogenesis regulates cancer stem cell properties and the potential application of therapeutic agents targeting lipid metabolism in cancer.

## Materials and methods

### Cells and reagents

Human colon cancer cell lines DLD1 and HCT116 cells were cultured in DMEM and McCoy’s 5A supplemented with 10% fetal bovine serum (FBS, Sigma-Aldrich, MO, USA) and 1% penicillin–streptomycin, respectively. These cells were purchased from ATCC and authenticated using short tandem repeat (STR) DNA profiling in March 2016 (Genetica, OH, USA). Primary colon cancer Pt130 cells were established from patient-derived xenografts (PDX) as described previously^40^. Briefly, this cell line, obtained from a 63-year old male with moderately differentiated colonic adenocarcinoma, contains BRAF (V600E) and TP53 mutations and was authenticated using short tandem repeat (STR) DNA profiling as a unique cancer cell line (Genetica). Stable SREBP1, SREBP2 and SCAP knockdown cells were generated using lentivirus-based RNAi^40–42^. The shRNA targeting sequences for SREBP1 (also named SREBF1) are as the following: 5′-GCCATCGACTACATTCGCTTT-3′ (a) and 5′-CCAGAAACTCAAGCAGGAGAA-3′ (b); for SREBP2 (also named SREBF2): 5′-CCTCAGATCATCAAGACAGAT-3′ (a) and 5′-GACCTGAAGATCGAGGACTTT-3′ (b); and for SCAP: 5′-GCTCAACGGTTCCCTTGATTT-3′ (a) and 5′-GCTCTGGTGTTCTTGGACAAA-3′ (b). The non-targeting control shRNA lentivirus plasmid (MISSION, SHC002) was obtained from Sigma-Aldrich (MO, USA). Fatostatin and 25-HC were purchased from Sigma-Aldrich.

### Real-time PCR

Total RNA was isolated from human cancer cells or xenograft tumors using RNeasy kit (Qiagen, MD, USA). Equal amounts of RNA were used as templates for the synthesis of cDNA using High Capacity cDNA Reverse Transcription kit (Applied Biosysems, CA, USA). Real-time PCR reaction was performed using TaqMan probes specific for *SREBP1*, *SREBP2*, *LDLR*, *FASN*, *SCD*, *ACACA*, *HMGCS*, *HMGCR*, *LGR5*, *CD44*, *CD133*, *LGR5*, and *AXIN2* using StepOne Real-Time PCR system (Applied Biosysems). All values were normalized to the level of β-actin.

### Measurements of cellular fatty acid, cholesterol, and triglyceride levels

Equal numbers of colon cancer cells were seeded in 6-well plate in regular growth medium. After 16 h incubation, cells were lysed and extracted according to procedures specified by individual quantification kits. The Free Fatty Acid Quantification Kit, Cholesterol Quantification Kit (Sigma-Aldrich), and Triglyceride Colorimetric Assay Kit (Cayman, MI, USA) were used to quantify levels of cellular fatty acids, cholesterol, and triglyceride, respectively.

### Cell proliferation assay

To determine the rate of proliferation, equal numbers of cells were seeded into 12-well plates (2 × 10^4^ cells per well). The number of cells were counted daily for 4 days using a cell counter (Beckman-Coulter, Fullerton, CA, USA).

### Spheroid formation assay

To determine the self-renewal capacity of colon cancer cells, 1,000 single cells were seeded in non-adherent 24-well plates in StemPro hESC SFM medium supplied with 1% GlutaMAX, 2% StemPro hESC supplement, 1.8% BSA, 8 ng/ml FGF-basic, and 0.1 mM 2-mercaptoethanol (Thermo Fisher Scientific, MA, USA). After 6 days in culture, numbers of spheroids were counted under a light microscope.

### Seahorse extracellular flux analysis

The Seahorse XF96 Extracellular Flux Analyzer (Agilent, CA, USA) was used to measure the respiration activity of colon cancer cells. Cells were seeded at the density of 3 × 10^4^ cells per well in a XF96 plate ~16 h before the measurement. The glycolysis and mitochondrial stress test were performed according to manufacturer’s protocol. All Seahorse measurements were normalized to the protein contents in each well. The relative levels of glycolysis, glycolytic capacity, and glycolytic reserve were calculated based on ECAR data obtained in the glycolysis stress tests, whereas the relative levels of basal, maximal, ATP production-related respiration, and reserved mitochondrial capacity were calculated based on OCR data obtained in the Mito stress tests using Seahorse Wave software for XF analyzers.

To measure the rate of cellular FAO, cells were incubated overnight in Substrate-Limited Medium (DMEM with 0.5 mM glucose, 1.0 mM glutamine, 0.5 mM carnitine, and 1% FBS) in XF96 cell culture plates. Forty-five minutes prior to the beginning of OCR measurement, the cells were switched to the FAO Assay Medium (111 mM NaCl, 4.7 mM KCl, 2.0 mM MgSO_4_, 1.2 mM Na_2_HPO_4_, 2.5 mM glucose, 0.5 mM carnitine, and 5 mM HEPES). Etomoxir (40 µM) was added to one set of cells to reveal the amount of FAO-associated OCR. All Seahorse measurements were normalized to the protein concentrations in each well. The FAO-dependent respiration was calculated by subtracting OCR levels in ETO-treated cells from those in untreated cells.

### Western blot analysis

Colon cancer cells were harvested and detergent-solubilized cell lysates were obtained as described previously^41–44^. Equal amounts of cell lysates were resolved by SDS-PAGE and subjected to western blot analysis. The SREBP1 antibody was from BD Biosciences (CA, USA), whereas the SREBP2 antibody was from R&D Systems (MN, USA). Note that the SREBP antibodies recognize both the precursor and mature active forms of the respective proteins. The γ-tubulin antibody was from Sigma-Aldrich.

### Xenograft tumor formation

All animal procedures were done using protocols approved by the University of Kentucky Animal Care and Use Committee. Six to eight week-old NOD.Cg-*Prkdc*^*scid*^ *Il2rg*^*tm1Wjl*^/SzJ (NSG, The Jackson Laboratory) mice were used. Both male and female mice of equal numbers were included in each group. Control and SREBP knockdown Pt130 cells were collected in 50% Matrigel in DMEM and inoculated subcutaneously at 1 × 10^6^ cells per injection site. After 2 weeks, the tumor size was measured every 3 days with a caliper, and the tumor volume was defined as (longest diameter) × (shortest diameter)^2^/2. At the end of experiments, tumors were harvested, weighted and fixed in 10% buffered formalin. The paraffin embedded samples were prepared, and 5 µm sections were used for IHC staining. For tumor initiation assay, Pt130 cells (1000 cells/injection site) were injected subcutaneously in NSG mice. The number of tumors formed was determined 3 months post injection.

### Immunohistochemical (IHC) staining

Paraffin embedded xenograft tumor sections were deparaffinized, rehydrated, and treated with hydrogen peroxide. Antigen retrieval was performed using Dako Target Retrieval Solution (Agilent), and IHC staining was performed as previously described^40,42^. The anti-Ki67 antibody was from Dako (Agilent, CA, USA) and the cleaved caspase-3 antibody was from Cell Signaling. The stained sections were visualized using a Nikon Eclipse 80i upright microscope. Tumor sections from three different mice per group were stained and examined. In each stained section, numbers of Ki67 or cleaved caspase-3-positive cells were counted in seven randomly chosen images and the percentage of staining positive cells were quantified by normalizing to total numbers of cells. Results shown in Fig. 7 represent average results obtained in three mice.

### Statistical analysis

In experiments to assess the rate of cell proliferation, mRNA expression, fatty acid and triacylglycerol (TAG) levels, bioenergetic measurements, and tumor weight were summarized using bar or line graphs and pairwise comparisons between different conditions were carried out using two-sample *t*-tests. A linear mixed model was employed to compare slope of tumor volume growth curves over time between groups. The relative mRNA expression results shown in Figs. 1–3 and 7 represent average of three separate qPCR experiments with four replicates for each gene in each experiment. For the Seahorse experiments, eight replicates were included for each cell line in each experiment and the experiments were repeated three times. Results shown in Figs. 4–6 were from one representative experiment. All other experiments were repeated three times and results shown represent the average of three experiments.

For the Gene Set Enrichment Analysis (GSEA), RNA sequencing data were obtained from the TCGA CRC study. Correlations between expressions of SREBP1 (gene name: *SREBF1*) and SREBP2 (gene name: *SREBF2*) and the other genes were quantified by Spearman’s correlation coefficient. The genes were then ordered from highest to lowest based on the correlation coefficient. This ranked list was inputted into the GSEA Desktop Application^45^ to identify pathways that are associated with SREBP expression.

## Electronic supplementary material

Supplemental Figures
