# Supplementary material for: Downregulation of SREBP inhibits tumor growth and initiation by altering cellular metabolism in colon cancer
Source: Cell Death Dis. 2018 Feb 15;9(3):265. doi: 10.1038/s41419-018-0330-6 (PMC5833501; doi:10.1038/s41419-018-0330-6)

**Downregulation of SREBP inhibits tumor growth and initiation by altering cellular metabolism in colon cancer**

**Supplemental Figures**

**Figure S1. Knockdown of SREBP1 or SREBP2 inhibits expression of lipogenic genes in colon cancer cells.** (**a**) The real-time PCR analysis of SREBP1 and SREBP2 expression in control and knockdown HCT116 cells. Two different shRNA targeting sequences were used for knocking down SREBP1 or SREBP2. (**b**) Knockdown of SREBP1 and SREBP2 decreased the expression of genes related to fatty acid synthesis and metabolism in HCT116 cells. Data represent the mean ± SD (* p < 0.001 and # p < 0.05 compared to sh-Control).


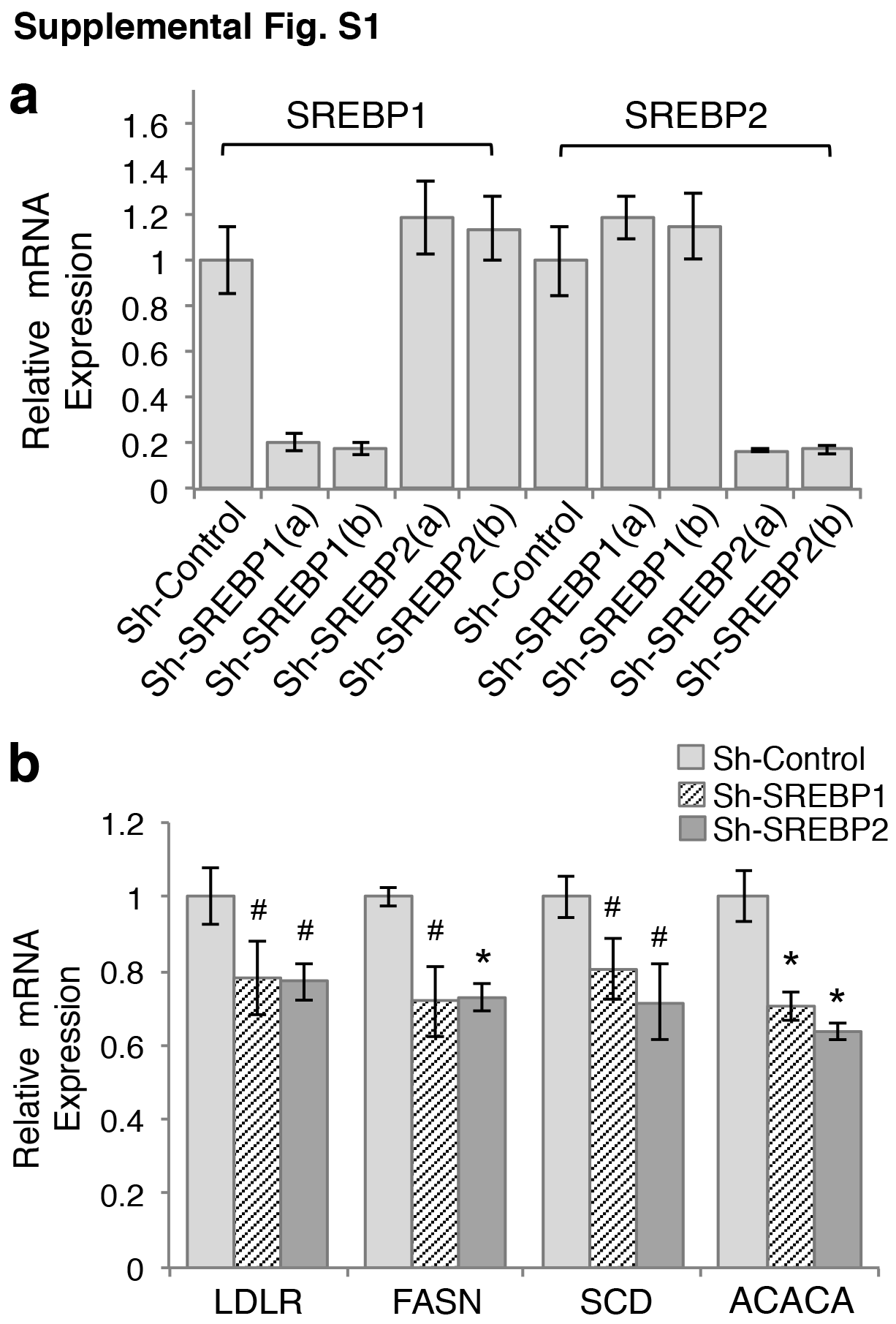


**Figure S2. Knockdown of SREBP1 or SREBP2 inhibits cell proliferation and tumor spheroid formation in colon cancer cells.** (**a**) Knockdown of SREBP1 or SREBP2 decreased the rate of proliferation in HCT116 cells. Equal number of control and SREBP knockdown cells were allowed to grow for 4 days and the number of cells were counted each day. Data represent the mean ± SD (* p < 0.001 and # p < 0.05 compared to sh-Control). (**b**) Knockdown of SREBP1 or SRBEP-2 decreased the formation of tumor spheroids in the stem cell suspension medium. Control and SREBP knockdown HCT116 cells were seeded as single cells in the stem cell suspension medium and the number of colonies formed was determined after 7 days. Data represent the mean ± SD (* p < 0.001 compared to sh-Control).


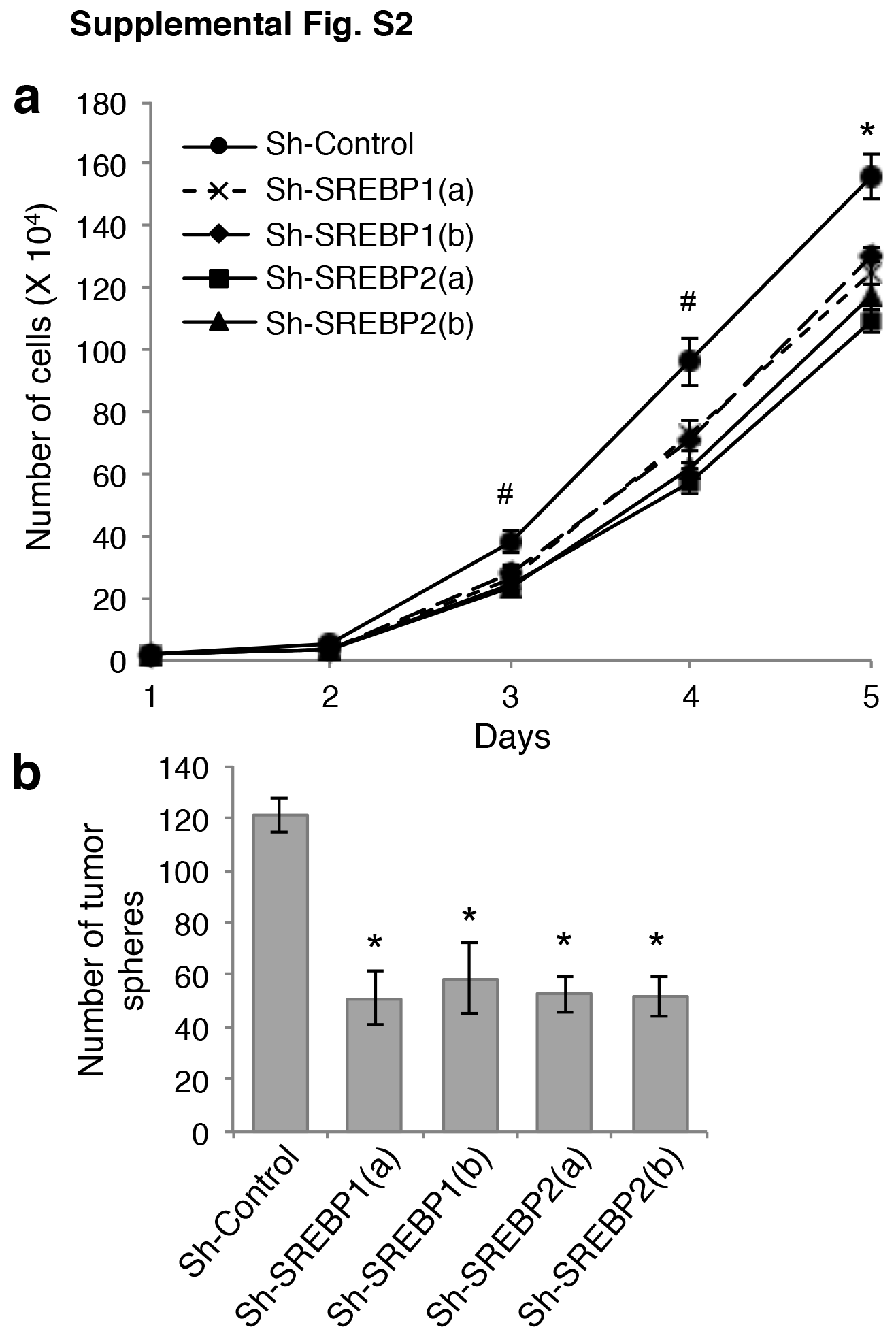


**Figure S3. Knockdown of SCAP inhibits cell proliferation and tumor spheroid formation in colon cancer cells.** (**a**) Knockdown of SCAP decreased the expression of genes related to fatty acid synthesis and metabolism in DLD1 and Pt130 cells. Data represent the mean ± SD (§ p < 0.01 and # p < 0.05 compared to sh-Control). (**b**) Knockdown of SCAP decreased the rate of proliferation in DLD1 and Pt130 cells. Data represent the mean ± SD (§ p < 0.01 and # p < 0.05 compared to sh-Control). (**c**) Knockdown of SCAP inhibited the formation of tumor spheroids in the stem cell suspension medium. Control and SCAP knockdown DLD1and Pt-130 cells were seeded as single cells in the stem cell suspension medium and the number of colonies formed was determined after 7 days. Data represent the mean ± SD (¶ p < 0.0001 compared to sh-Control). (**d**) Knockdown of SCAP reduced the expression of genes associated with colon cancer stem cells. The relative expression of CD44, CD133, LGR5 and Axin2 mRNA was determined using real-time PCR in control and SCAP knockdown DLD1 and Pt130 cells. Data represent the mean ± SD (§ p < 0.01 and # p < 0.05 compared to sh-Control).


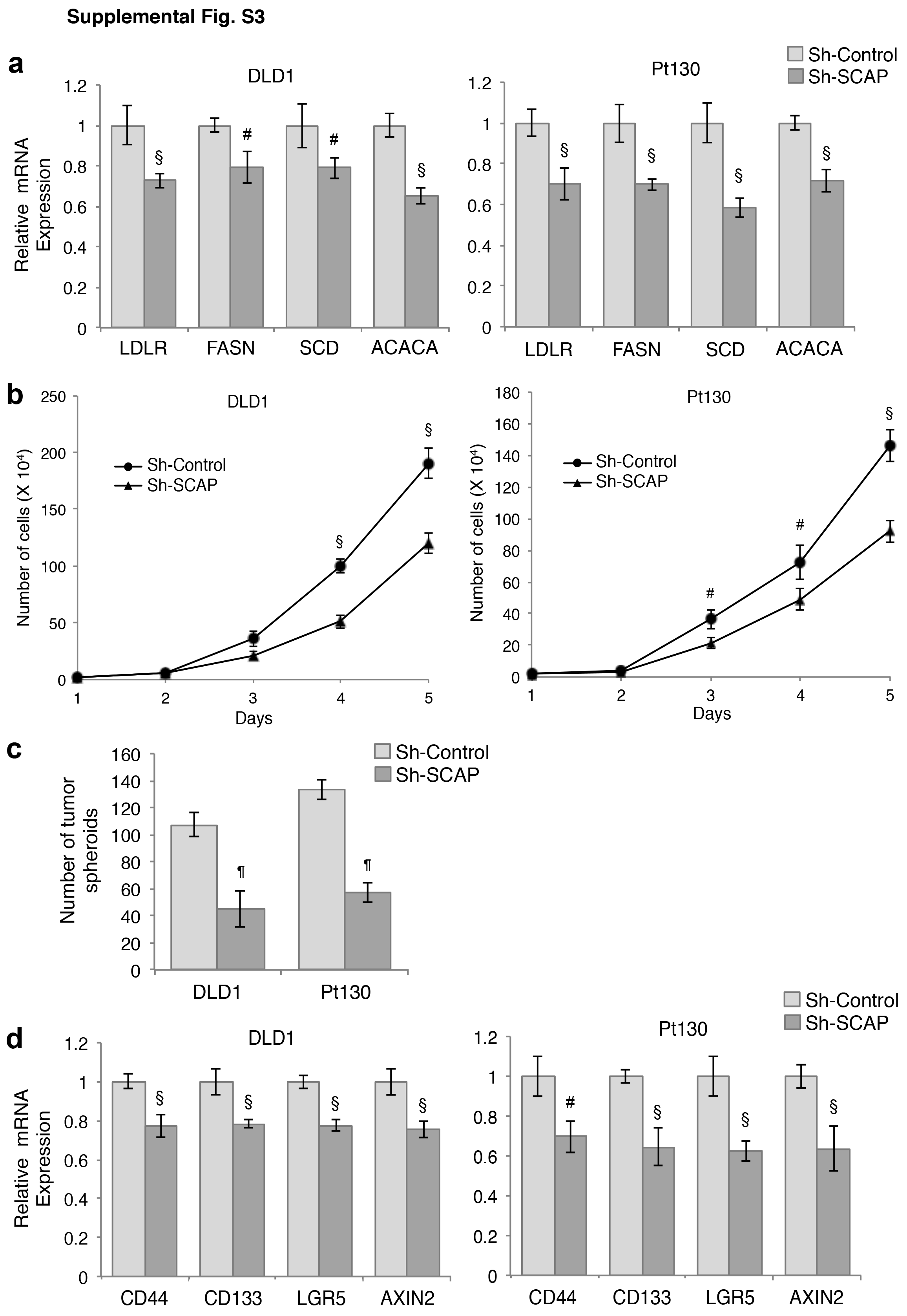


**Figure S4. Gene Set Enrichment Analysis (GSEA) of SREBP expression in CRC patients.** TCGA CRC RNA-seq dataset was first used to identify genes that have positive correlations with SREBP1 (gene name: *SREBF1*) and SREBP2 (gene name: *SREBF2*) expression. The GSEA was then performed to determine if *SREBF1* and *SREBF2* expression is associated with gene sets in the REACTOME and KEGG collections. The name of the gene sets and the corresponding normalized enrichment score (NES) and false discovery rate (FDR) are listed in the table (the cutoff for significance is set for FDR < 0.05).


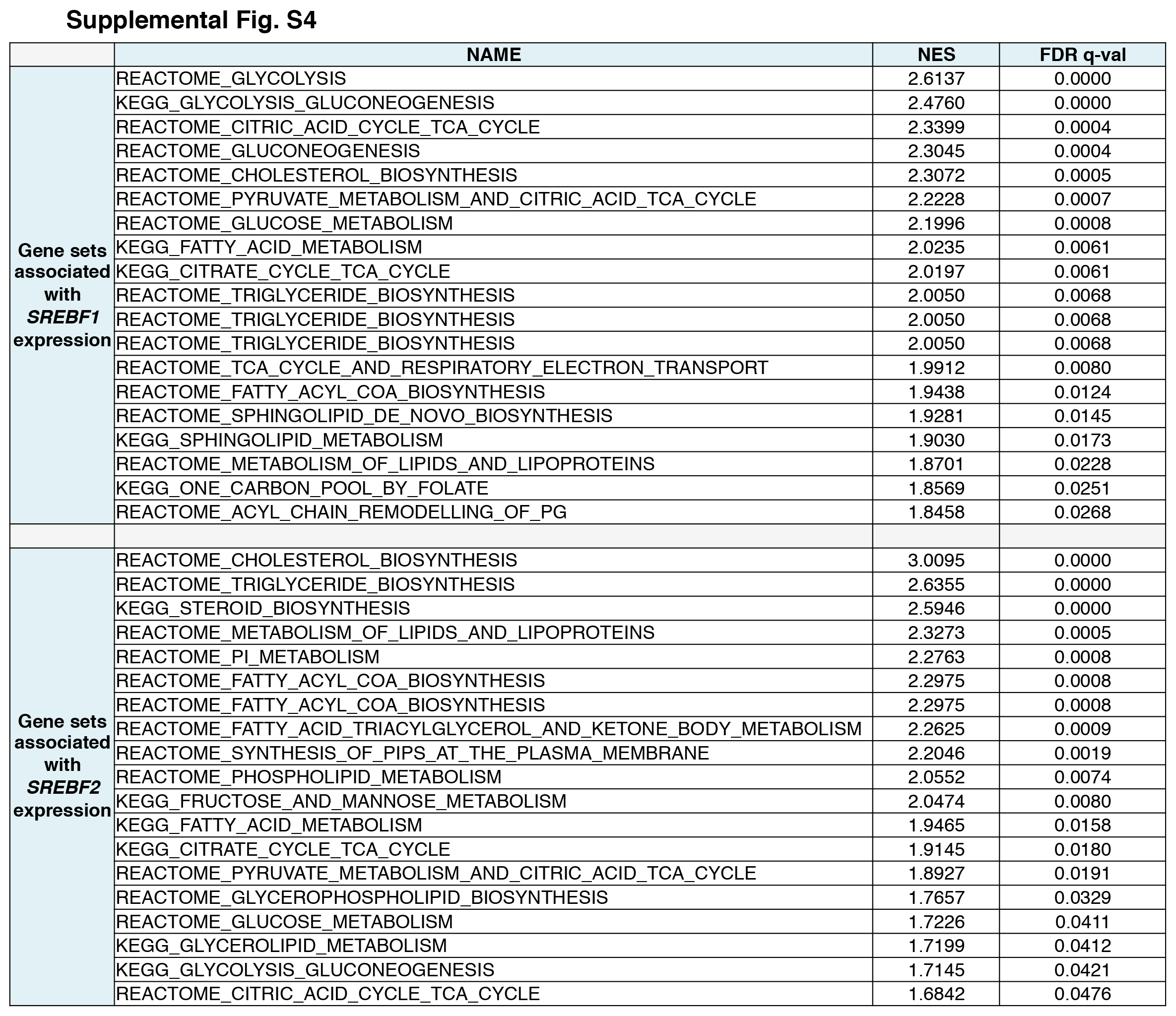

Supplement: Supplementary file 1 — Supplemental Figures [file 41419_2018_330_MOESM1_ESM.docx]
